# Frequency of Iron Deficiency Anemia in Girls Studying in Mashhad High Schools

**Published:** 2013-10-22

**Authors:** F Abrishami, A Golshan

**Affiliations:** 1 Faculty of Medicine, Ghaem Hospital, Mashhad University of Medical Sciences and Health Services, Mashhad, Iran.

**Keywords:** Anemia, Iron deficiency, Malnutrition

## Abstract

**Background:**

Iron deficiency is one of the most prevalent anemia. 2 million people in the world suffer from it. All young girls are at higher risk for iron defiency anemia, therefore,diagnosis and prevention of this anemia in the young age is very important.

**Materials and Methods:**

A total of 1500 high school girls educated in five regions of education of Mashhad (ages 14-18 years) were studied. Cell blood count (CBC), serum iron, total iron binding capacity(TIBC),ferritin and peripheral blood smear were performed .

If mean corpuscular volume (MCV) was less than normal(<76fl) and Red blood cell (RBC) was more than normal(>5×106/mm3 ), hemoglobin electrophoresis was subjected to test by methods of cellulose acetate to check the possibility of thalassemia minor.The data was analyzed by SPSS(version19) and Minitab software.

**Result:**

This is a descriptive cross sectional research. From 1500 under-experiment people,1094 cases (72.9%) were non-infected, 310 cases(20.7%) had iron deficiency anemia, and 96 cases(6.4%) had other disorders such as thalassemia.

In girls with anemia, 272 cases (87.7%) were in stage I, 17 cases (5.5%) in stage II and 21 cases (6.8%) in stage III. The average age in stage I was higher than stage II and III. . Mean and standard deviation for Hb, Hct, MCV, MCH, MCHC, Fe, TIBC and Ferritin had significant difference in infected and non-infected group.

**Conclusion:**

This study revealed that the prevalence of iron deficiency anemia in young girls are moderate, so that it is important to reduce the prevalence of iron deficiency anemia in young girls.

## Introduction

Anemia is a condition that develops when the blood Anemia - caused by lack of nutrients - is considered to be one of the most prevailing outcomes of insufficient diets from which nowadays human being suffer. According to World Health Organization in Eastern Mediterranean Region there are nearly 149 million people suffering from anemia (1). Anemia has different reasons, but almost 50 percent of anemia occur due to iron deficiency (2). Iron deficiency anemia is the most prevailing anemia in the world and more than 2 billion people are infected with this disease (3). Iron is considered to be an essential micro-nutrient for proper function of body organs and for normal learning, positive behavioral changes, suitable cognitive reaction and metabolism of hormones and reproduction (4-5). Also during the puberty, due to growth process, the body requires more iron (6). The side-effects of iron deficiency include: decrease of working capacity, decrease of reproduction strength, decrease of memory function, decrease of learning potentiality and subsequently academic failure during the school time and decrease of endurance against diseases caused by weakness of immune system (7-8-9). Prevalence of iron deficiency anemia in underdeveloped countries is more than developed countries (10). Prevalence of this disease among 15 to 59 year-old-women in more developed countries is 10.3% while in non-industrial countries it is 42.3% (11). Also women are significantly more exposed to iron deficiency anemia than men. The studies show that iron deficiency and anemia has been observed in girls 10 times more than boys (12). A study conducted in Thailand during 2006 indicated that the prevalence of this disorder in pregnant women is equal to 37.8% (8). According to reports, prevalence of anemia, iron deficiency and the anemia caused by iron deficiency in Kermanshah-female-high school students are equal to 21.4%, 23.7% and 12.2% respectively (13). In girls - due to initiation of their menstrual period - the individual is more exposed to iron deficiency; this is because the teenage loses so much iron due to her menstrual period. The consequence of this anemia during the puberty has negative effects on fertility of the individual in future and has the risks of: delivery of low birth weight, preterm birth, and abortion; also it has been reported that 20% of maternal mortality has been due to iron deficiency (14). Also some research conducted in teenage girls indicated that iron deficiency anemia may lead to decrease of physical strength, behavioral changes and decrease in concentration ability (15). Due to the importance of these problems and the subsequent side-effects of iron defiency on body, mind and the society, this research was conducted to study the prevalence of iron deficiency in 1500 girls studying in Mashhad high schools.

## Materials and Methods

The study has been approved by the Ethic Committee This descriptive cross sectional research has been conducted during 12 months (2007-2009) on 1500 girls, aged between 14 to 18 years-old, whom studied in 10 high schools in Mashhad from five regions. In this study, after receiving certificates from teaching and training organization of Khorasan Razavi, in order to conduct the research, required coordination were made with school managers; afterwards the statistics of female-students studying in first, second and third grade of high school as well as pre-university grade were acquired from teaching and training organization. 1500 students were determined based on population of regions of the city. From each region of the city two high schools were randomly selected and Due to population of the classes some students were chosen as cluster Random sampling. 

The sampling was started with taking 5cc of blood. 1cc was kept in tubes containing EDTA for measuring CBC, Hb , Het ,MCV ,MCH and MCHC and the rest of the blood was kept in test tube. Immediately after sampling, expansion of peripheral blood was provided and it was stained by Giemsa solution. Reticulocyte coloring was also conducted on samples and serum iron, total iron binding capacity (TIBC) (by biochemistry method) and ferritin(by ELISA method with DRG Ferritin kit) experiments were also conducted on blood serum of the samples. If mean corpuscular volume (MCV) was less than normal (<76fl) and Red blood cell (>RBC) was more than normal(5×106/mm3 ), hemoglobin electrophoresis was subjected to test by methods of cellulose acetate and citrate agar to check the possibility of thalassemia minor.

 The criterion of iron deficiency in Stage I ,II ,III was as follow: 

Stage I: ferrtin<10ng/ml (16-17).

Stage II: ferrtin<10ng/ml ,RDW>14.5 ,RBC<4.2×1012/L ,Fe<50µg/dl ,TIBC>450µg/dl (16-17).

Stage III: ferrtin<10ng/Ml, RDW>14.5, RBC<4.2×1012/L, Fe<50µg/dl, TIBC>450µg/dl, Hb<12gr/dl, MCV<76fl (16-17) . 

## Results

**Figure 1:**
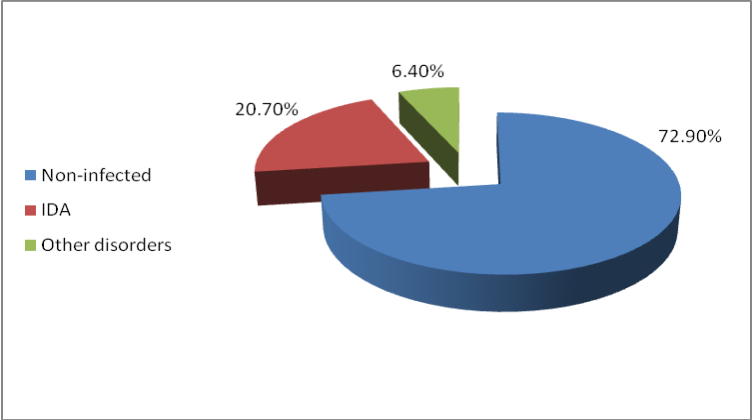
Prevalence of iron deficiency anemia in girls studying in Mashhad high schools

From 1500 under-experiment people,1094 cases (72.9%) were non-infected, 310 cases(20.7 )had iron deficiency anemia, and 96 cases(6.4%) had other disorders such as thalassemia, spherocytosis (Figure 1).

**Figure 2:**
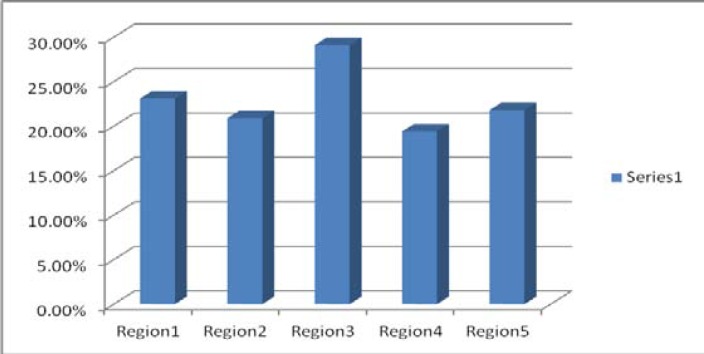
Prevalence of iron deficiency anemia in different regions

Prevalence of iron deficiency anemia in different regions was as follows: 

Region1 (23.1%), region 2 (20.9%), region3 (29.1%), region 4 (19.4%) and region 5 (21.8%). As it is seen, the maximum prevalence of IDA was in region 3 (29.4%) and the minimum was in region 4 (19.4%). According to Chi-square test, the hypothesis of independence of the regions will not be rejected by anemia; in other words, different regions did not have any effect on anemia. (Figure 2).

**Figure 3:**
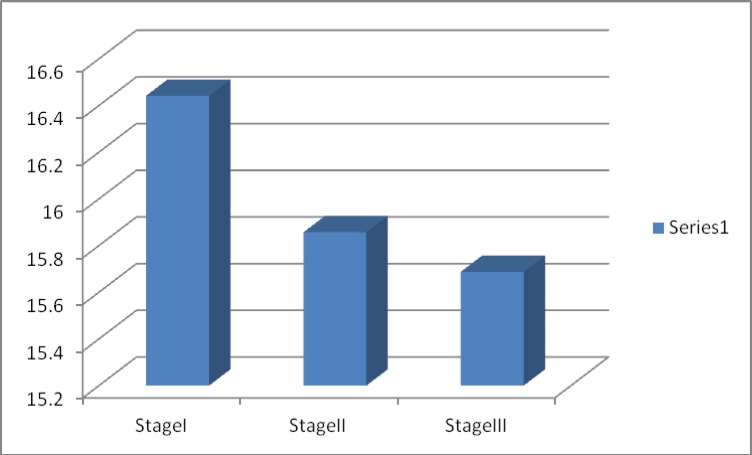
Average age in stageI , II and III

 The prevalence of anemia in different high schools was studied and the maximum prevalence was observed in Nemune Andishe high school (34.6%) and the minimum was in Tadayon high school (18.1%). According to Chi-square test, there is a significant difference between each high schools and iron deficiency anemia with confidence of 90-95 percent. From 310 people who were suffering from iron deficiency anemia, 272 (87.7%) people were categorized in stageI, who had lower ferritin than the normal, but other criteria were normal. Also 17(5.5%) people are categorized in stageII, which means ferritin, serum iron, and the saturation percentage of transferrin are lower than normal in them and also TIBC and RDW was more than normal in this group, but other blood findings was normal. Also 21(6.8 %) people were categorized in stage III, which means all blood findings were abnormal in them and they were suffering from Microcytic hypochromic anemia. There was an obvious difference between the average age of individuals in stage I, II and III. It means that the average age in stage I, is higher than stages II and III (Figure 3).

Mean and standard deviation of hemoglobin, hematocrit, MCV, MCH, iron, TIBC and ferritin in the two groups of infected and non-infected people had significant difference with 95% confidence. There was no significant difference between RBC of infected and non-infected people. The effect of some of the factors influencing the occurrence of this disease has been analyzed separately.

**Figure 4:**
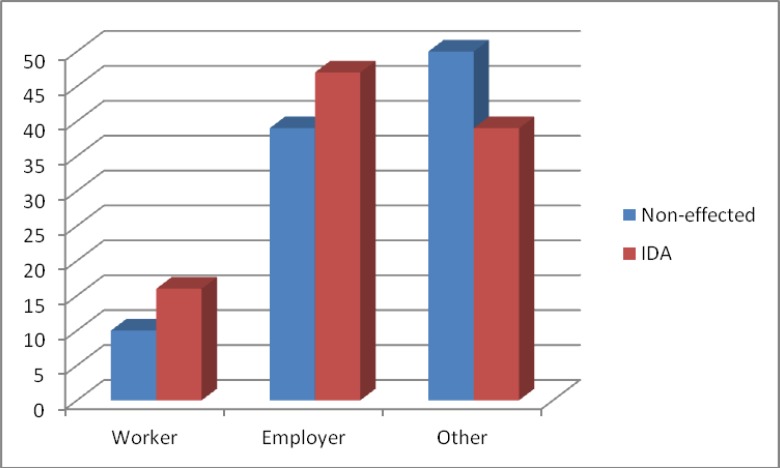
The prevalence of anemia due to the job of father

The highest prevalence of iron deficiency anemia was observed in the children of employee-class family and the lowest level was observed in the children of non-employee category family (Figure 4). But there was no correlation between parental education level and iron deficiency. Also the highest prevalence of IDA was observed in families with average income of less than 800 dollars, and the lowest rate was observed in families with average income of 1600 dollars. The average of the number of family members had no apparent difference in infected and non- infected groups.

## Discussion

The results of the present study which has been conducted in order to consider the prevalence rate of iron deficiency anemia in 1500 girls of high schools in Mashhad, showed that out of 1500 studied girls 1094 girls (72.9%) were non-infected 310 girls (20.7%) were suffering from iron deficiency anemia, and 96 girls (6.4%) had other disorders such as thalassemia, ovalocytosis. Out of 310 suffering people, 87.7% of the girls were in stage I, 5.5% in stage II and 6.8% in stage III. Mean and standard deviation of hemoglobin, hematocrit, MCV, MCH, iron, TIBC and ferritin in the two groups of infected and non-infected people had a significant difference. Factors such as the age of the patient, their parents' job, and the income of their parents had considerable effect on the IDA rate. The average age of girls in stage 1 was higher than stage II and III.

According to estimations in Eastern Mediterranean region, about 149 million people are infected by anemia based on the criteria of the World Health Organization. The results of a study which was conducted in 2012 indicate that the prevalence rate of anemia among studied people is 59.1% which signifies a high percentage (18). Also, another study in 2011 reported that the prevalence rate of anemia among the studied students is 34% (19).

According to the definition presented by the World Health Organization based on the prevalence rate of anemia in the region, the severity of the problem is defined as a public health problem, so that prevalence less than 5% is not considered as public health problem, but prevalence of 5 to 19.9, 20 to 39.9 and more than 40 percent is considered as minor, average, and major problems of public health respectively. Accordingly, considering the results of present study, this problem is evaluated as average (20).

Iron deficiency anemia is the most prevalent anemia, and usually comprises 50 percent of anemias. In some of the studies, 88.4% of anemias are comprised of iron deficiency anemia (21). In Iran, due to the differences in diet, culture and lifestyle of people, the prevalence rate of iron deficiency anemia in different regions of the country is reported very differently. Among the most important factors of iron deficiency anemia we can refer to inadequate intake of iron through diets, parasitic infections with hookworms, gastrointestinal diseases, growth spurt, period, and pregnancy (22). In a study in 2008 with the purpose of surveying iron deficiency anemia in women of childbearing age in Ramsar and Tonekabon cities, it is reported that the prevalence rate of iron deficiency anemia is 21.3% (23). Moreover, a study has been conducted in Indonesia on young girls in order to consider the prevalence rate of iron deficiency anemia. This study showed that 21.8% of the studied people have this type of anemia (24). The results of these two studies were to some extent in accordance with our reported results. In the present study, the prevalence rate of iron deficiency anemia is 20.7%. In another study during the years 2007 to 2008 with the purpose of considering prevalence of iron deficiency and its resulting anemia among high school girls of Yazd city, it has been reported that generally 13.5% of the studied people had anemia out of which 9.3% had iron deficiency anemia (25). In a study conducted in 2002 in the United Sates of America, the prevalence rate of iron deficiency has been reported 2% (26). This rate is much less than the present study, which may possibly be due to better social, economical and nutritional status in their research.

In the present study, there was a significant relationship between prevalence of iron deficiency anemia, job and income of the father of the family, in a way that the highest prevalence of iron deficiency anemia was observed in the children with employee-class families and the lowest level was observed in the children with Non-employee category families. Also the highest prevalence of IDA was observed in families with average income of less than 800 dollars and the lowest rate was observed in families with average income of s1600 dollars. In a study in Saudi Arabia it was observed that iron deficiency anemia was more prevalent in students whose mothers’ educational level was lower(27). In a study which has been conducted on the students of guidance schools of Birjand city in 2006, there was no significant relationship between prevalence of iron deficiency sanemia, age, job, and education level of parents (28).

## Conclusion

In the present study, because the prevalence of iron deficiency anemia is moderate, it seems to be important to organize the politics to reduce the prevalence of iron deficiency anemia.

## Conflict of interest

The authors have no conflict of interest.
